# Supplementary material for: Experimental realization of an extended Fermi-Hubbard model using a 2D lattice of dopant-based quantum dots
Source: Nat Commun. 2022 Nov 11;13:6824. doi: 10.1038/s41467-022-34220-w (PMC9652469; doi:10.1038/s41467-022-34220-w)
Supplement: Supplementary file 1 — Supplementary Information [file 41467_2022_34220_MOESM1_ESM.pdf]

Supplementary Information

Experimental Realization of an Extended Fermi-Hubbard Model Using a 2D Lattice of Dopant-based Quantum Dots

Xiqiao Wang,[1,2,^] Ehsan Khatami,[3] Fan Fei,[1,4] Jonathan Wyrick,[1] Pradeep Namboodiri,[1] Ranjit Kashid,[1,#] Albert F. Rigosi,[1] Garnett Bryant,[1,2] Richard Silver[1*]

[1] Atom Based Device Group, National Institute of Standards and Technology, Gaithersburg, MD 20899

[2] Joint Quantum Institute, University of Maryland, College Park, MD 20740

[3] Department of Physics and Astronomy, San José State University, San José, CA 95192

[4] Department of Physics, University of Maryland, College Park, MD 20740

[^] Current address: Rigetti Computing, Fremont, CA 94538

[#] Current address: Center for Materials for Electronics Technology, Pune 411008, India

[*] Corresponding author Richard.silver@nist.gov

Supplementary Note 1. Capacitance modeling of the 3x3 array devices

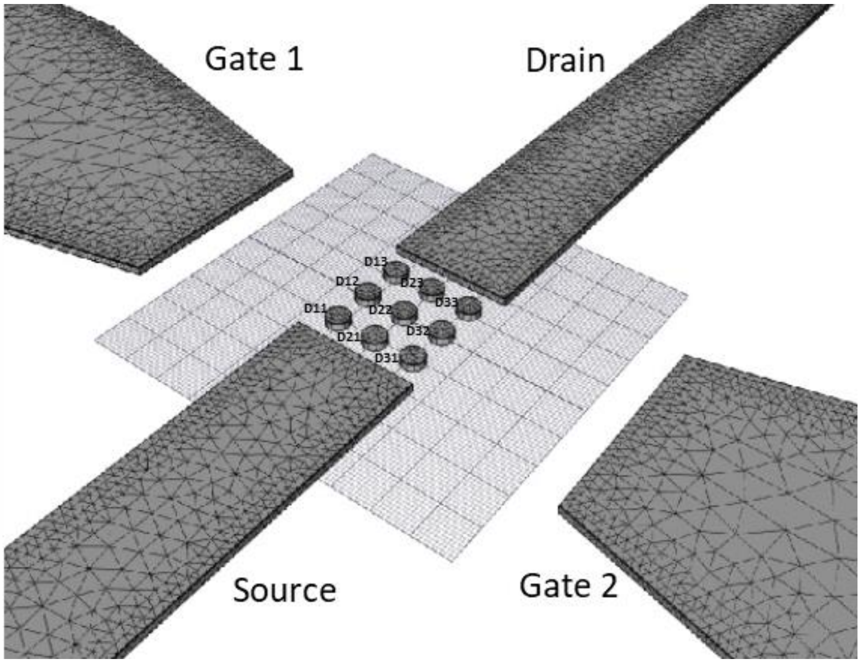

Supplementary Fig. 1. Classical capacitance model of the 3x3 array buried in the device geometry. The figure shows a perspective view of the mesh geometry of the first array for modeling the capacitance matrix of the device.

Gate-tunability of the chemical potential landscape within the array is achieved via classical capacitive coupling between the two in-plane gates and the lattice site in the array. From the equivalent circuit model under the constant interaction approximation,[1] there exists constant capacitive coupling between the electrons at a lattice site i and a metallic electrode l, denoted as $C_{l,i}$, and between electrons at different lattice sites, denoted as $C_{i,j}$. Here $l \in [D, S, G1, G2]$ for the drain, source, gate1, and gate2 electrodes, and $i, j \in [11,12,13,21,22,23,31,32,33]$ for the quantum dot sites from $D11$ to $D33$. The total capacitance of site i is $C_{\Sigma i} = \sum_l C_{l,i} + \sum_{j\neq i} C_{i,j}$. Therefore, the capacitance matrix of the array system can be expressed as,

$$\hat{C} = \begin{bmatrix} C_{\Sigma 11} & -C_{11,12} & -C_{11,13} & -C_{11,21} & -C_{11,22} & -C_{11,23} & -C_{11,31} & -C_{11,32} & -C_{11,33} \\ -C_{11,12} & C_{\Sigma 12} & -C_{12,13} & -C_{12,21} & -C_{12,22} & -C_{12,23} & -C_{12,31} & -C_{12,32} & -C_{12,33} \\ -C_{11,13} & -C_{12,13} & C_{\Sigma 13} & -C_{13,21} & -C_{13,22} & -C_{13,23} & -C_{13,31} & -C_{13,32} & -C_{13,33} \\ -C_{11,21} & -C_{12,21} & -C_{13,21} & C_{\Sigma 21} & -C_{21,22} & -C_{21,23} & -C_{21,31} & -C_{21,32} & -C_{21,33} \\ -C_{11,22} & -C_{12,22} & -C_{13,22} & -C_{21,22} & C_{\Sigma 22} & -C_{22,23} & -C_{22,31} & -C_{22,32} & -C_{22,33} \\ -C_{11,23} & -C_{12,23} & -C_{13,23} & -C_{21,23} & -C_{22,23} & C_{\Sigma 23} & -C_{23,31} & -C_{23,32} & -C_{23,33} \\ -C_{11,31} & -C_{12,31} & -C_{13,31} & -C_{21,31} & -C_{22,31} & -C_{23,31} & C_{\Sigma 31} & -C_{31,32} & -C_{31,33} \\ -C_{11,32} & -C_{12,32} & -C_{13,32} & -C_{21,32} & -C_{22,32} & -C_{23,32} & -C_{31,32} & C_{\Sigma 32} & -C_{32,33} \\ -C_{11,33} & -C_{12,33} & -C_{13,33} & -C_{21,33} & -C_{22,33} & -C_{23,33} & -C_{31,33} & -C_{32,33} & C_{\Sigma 33} \end{bmatrix}$$

Supplementary Eq. (1)

The electrostatic charge at site i can be expressed as $q_i = -n_i e + \sum_l C_{l,i} V_l$, where $n_i \in [0,1,2]$ is the integer number of electrons added onto the site via hopping, and $C_{l,i} V_l$ is the capacitively induced charge when applying a voltage V_l on electrode l. Here, e is the absolute charge value of an electron; and we neglect the background induced charges from the substrate. The classical charge at the array can be expressed as,

$$\hat{Q} = \begin{bmatrix} q_{11} \\ q_{12} \\ q_{13} \\ q_{21} \\ q_{22} \\ q_{23} \\ q_{31} \\ q_{32} \\ q_{33} \end{bmatrix}$$

Supplementary Eq. (2)

The electrostatic potential $\hat{V}$ and the total electrostatic energy P of the array can be obtained from,

$$\hat{V} = \frac{\hat{Q}}{\hat{C}}$$

$$P = \frac{1}{2} \hat{V} \cdot \hat{Q}$$

Supplementary Eq. (3)

Here P is a function of integer numbers of electrons at each site n_i and the voltage values at each electrode V_l. When $n_i = 0$ for all sites, we denote the total electrostatic energy as P_0. At a given set of voltage conditions, we denote p_i and U_i as the electrostatic chemical potential and on-site charging energy to add the first and second electrons, respectively, onto site i, which can be numerically calculated from,

$$p_i = P(0, \ldots, n_i = 1, \ldots, 0) - P_0$$

$$U_i = [P(0, \ldots, n_i = 2, \ldots, 0) - P(0, \ldots, n_i = 1, \ldots, 0)] - p_i$$

Supplementary Eq. (4)

The electrostatic mutual charging energy $U_{i,j}$ between site i and site j can be calculated from,

$$U_{i,j} = \left[P\left(0, \ldots, n_i = 1, \ldots, 0, \ldots, n_j = 1, \ldots, 0\right) - P_0\right] - p_i - p_j$$

Supplementary Eq. (5)

Note, it can be numerically verified that U_i and $U_{i,j}$ are independent of V_l. On the other hand, U_i and $U_{i,j}$ depend critically on the size of the metallic disk that is used to represent a quantum dot in the classical capacitance model. In each of the array devices in this study, we chose the disk size so that the average value of U_i (see Supplementary Table 1(a)) matches (within +/-2meV range) the average

expected on-site charging energy values of few-dopant clusters from previous atomic tight-binding calculations (See the last column in Supplementary Table 3).

We denote $\alpha_{l,i}$ as the lever arm of an electrode l to a lattice site i, which is defined as the ratio between $\Delta p_{l,i}$, the shift in the electrostatic chemical potential at site i in response to $\Delta V_l \times e$, the change in the electrostatic potential at electrode l.

$$\alpha_{l,i} = \frac{\Delta p_{l,i}}{\Delta V_l \times e}$$

Supplementary Eq. (6)

As described in the main text, the gate-gate slope at a charge addition boundary corresponds to the gate-gate level arm ratio of the added electron, that is, the conducting electron, and characterizes whether the single electron is added onto sites in the upper, middle, or lower row in the array. Here we define the effective lever-arm of gate l to an $(N+1)th$ addition-electron, which, with finite hopping, has the charge distribution $\sum_{i,\sigma} \Delta n_{i,\sigma}$, as,

$$\alpha_l(N \to N+1) = \sum_i \Delta n_i \cdot \alpha_{l,i}$$

Supplementary Eq. (7)

Here Δn_i represents the change in occupation at site i when a single electron is added onto the array, altering the charge distribution of the array from an eigenstate of a total charge number N to that of an eigenstate of $N+1$. The charge number conservation condition requires that $\sum_i \Delta n_i = 1$. On a numerically simulated charge stability diagram, we can extract the added electron occupations at charge addition boundaries, see plots in Fig. 3c in the main text, for instance, and calculate the lever arm ratio $\frac{\alpha_{G1,N\to N+1}}{\alpha_{G2,N\to N+1}}$ at each pixel point. In the right panels in the main text Fig. 4, we plot the histogram distributions of the lever arm ratio calculated from the simulated charge stability diagrams for the first, second, and third arrays, respectively.

On a conductance map spanned by voltages of the two in-plane gates, the gate-gate lever arm ratio equals the slope of the conduction line $\frac{\alpha_{G1}}{\alpha_{G2}} = \frac{-\Delta V_{G2}}{\Delta V_{G1}}$. Indeed, we observe qualitative agreement between the measured slope histograms and calculated lever arm ratio histograms in the main text Fig. 4.

(a)

On-site electron-electron Coulomb repulsion U_i (meV)			
	First Array	Second Array	Third Array
U_{11}	46.81	46.95	45.57
U_{12}	45.81	47.89	47.95
U_{13}	46.93	46.95	45.57
U_{21}	44.14	45.61	44.66
U_{22}	42.97	46.32	47.33
U_{23}	44.15	45.61	44.66

U_{31}	46.26	46.95	45.57
U_{32}	45.23	47.89	47.95
U_{33}	46.00	46.95	45.57

(b)

Long-range electron-electron Coulomb repulsion $U_{i,j}$ (meV)			
	First Array	Second Array	Third Array
$U_{11,12}$	20.25	13.78	8.19
$U_{11,13}$	10.92	5.94	2.89
$U_{11,21}$	19.06	13.77	6.72
$U_{11,22}$	13.94	9.01	4.82
$U_{11,23}$	9.20	4.80	2.14
$U_{11,31}$	10.08	6.05	2.30
$U_{11,32}$	9.10	5.45	2.42
$U_{11,33}$	6.94	3.47	1.36
$U_{12,13}$	20.27	13.78	8.19
$U_{12,21}$	13.96	9.02	4.77
$U_{12,22}$	19.06	15.00	8.90
$U_{12,23}$	14.02	9.02	4.77
$U_{12,31}$	9.08	5.45	2.42
$U_{12,32}$	10.71	7.16	3.50
$U_{12,33}$	9.10	5.45	2.42
$U_{13,21}$	9.09	4.80	2.14
$U_{13,22}$	13.88	9.01	4.82
$U_{13,23}$	19.15	13.77	6.72
$U_{13,31}$	6.89	3.47	1.36
$U_{13,32}$	9.07	5.45	2.42
$U_{13,33}$	10.10	6.05	2.30
$U_{21,22}$	18.68	13.05	7.89
$U_{21,23}$	10.35	5.66	2.60
$U_{21,31}$	18.86	13.77	6.72
$U_{21,32}$	13.79	9.02	4.77
$U_{21,33}$	9.04	4.80	2.14
$U_{22,23}$	18.67	13.05	7.89
$U_{22,31}$	13.74	9.01	4.82
$U_{22,32}$	18.85	15.00	8.90
$U_{22,33}$	13.73	9.01	4.82
$U_{23,31}$	8.98	4.80	2.14
$U_{23,32}$	13.78	9.02	4.77
$U_{23,33}$	18.85	13.77	6.72
$U_{31,32}$	19.79	13.78	8.19
$U_{31,33}$	10.65	5.94	2.89
$U_{32,33}$	19.72	13.78	8.19

(c)

	Gates' lever arms to each quantum dot $\alpha_{l,i}$								
	First Array			Second Array			Third Array		
	$\alpha_{G1,i}$	$\alpha_{G2,i}$	$-\alpha_{G1,i}$ $/\alpha_{G2,i}$	$\alpha_{G1,i}$	$\alpha_{G2,i}$	$-\alpha_{G1,i}$ $/\alpha_{G2,i}$	$\alpha_{G1,i}$	$\alpha_{G2,i}$	$-\alpha_{G1,i}/\alpha_{G2,i}$
Dot11	0.096	0.093	-1.042	0.158	0.101	-1.563	0.153	0.084	-1.834
Dot12	0.103	0.099	-1.043	0.175	0.116	-1.514	0.186	0.107	-1.746
Dot13	0.097	0.093	-1.041	0.158	0.101	-1.563	0.153	0.084	-1.834
Dot21	0.082	0.102	-0.806	0.121	0.121	-1.000	0.105	0.105	-1.000
Dot22	0.087	0.109	-0.799	0.138	0.138	-1.000	0.136	0.136	-1.000
Dot23	0.082	0.102	-0.802	0.121	0.121	-1.000	0.105	0.105	-1.000
Dot31	0.073	0.119	-0.610	0.101	0.158	-0.640	0.084	0.153	-0.545
Dot32	0.077	0.128	-0.605	0.116	0.175	-0.661	0.107	0.186	-0.573
Dot33	0.073	0.121	-0.600	0.101	0.158	-0.640	0.084	0.153	-0.545

Supplementary Table 1. Calculated on-site electron-electron interactions (U_i), long-range electron-electron interactions ($U_{i,j}$), and gate lever arms ($\alpha_{l,i}$) in the three arrays presented in this study.

Supplementary Note 2. Image analysis of STM-patterned quantum dots

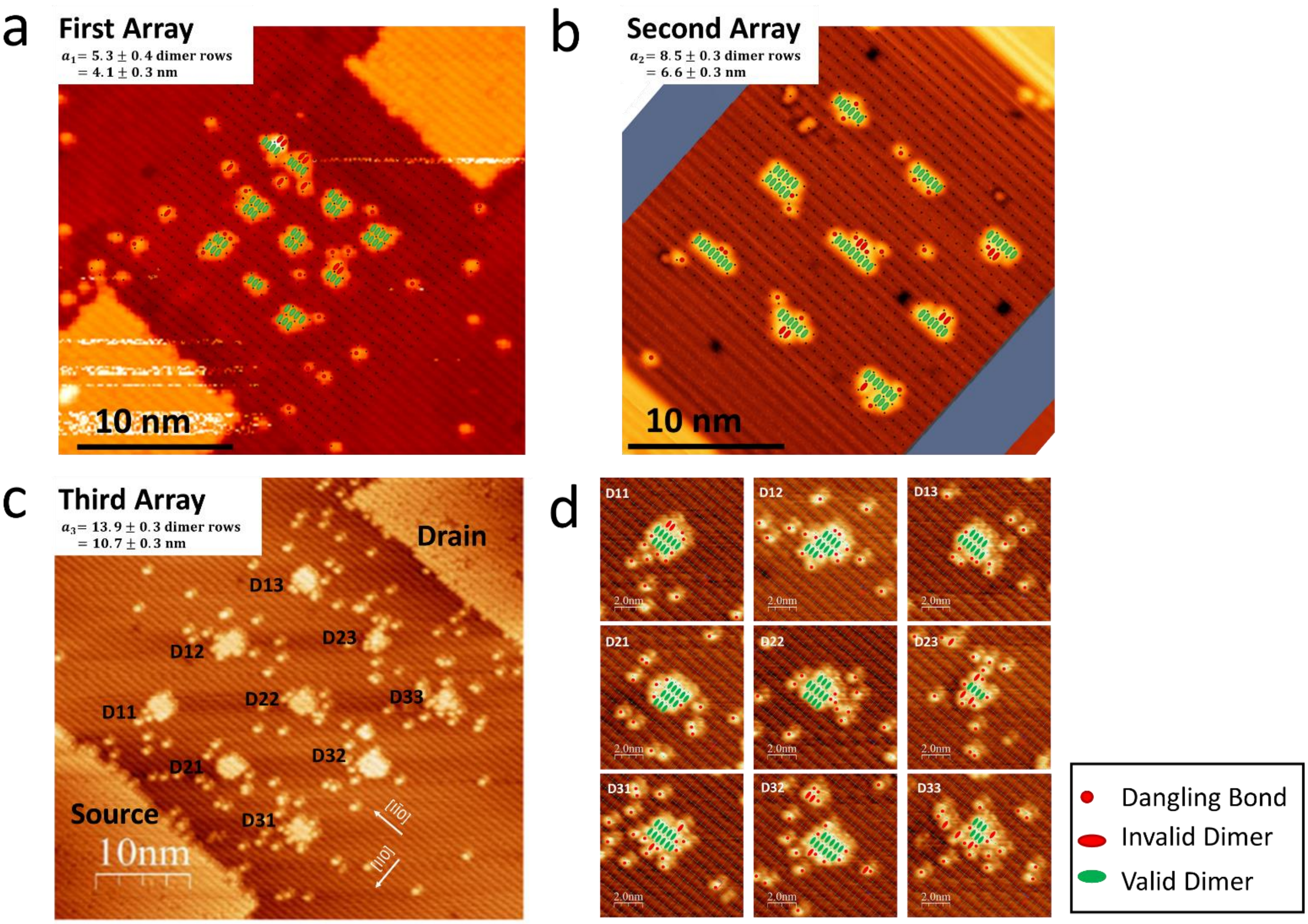

Supplementary Fig. 2. Estimating the number of incorporated dopant atoms at each quantum dot based on atomic-resolution STM images of hydrogen lithography patterns before phosphine dosing. (a) (b) (c) Atomic resolution STM image of the central region of the first array device (a), the second array device (b), and the third array device (c) after hydrogen lithography but before phosphine dosing, overlayed with surface lattice grids and identifiers of desorbed dangling bonds and dimers at each array site. (d) Zoom-in images at each quantum dot in (c) with overlayed grids of Si(100) 2x1 surface reconstruction unit cells and identifiers of desorbed dangling bonds and dimers at each array site.

At each quantum dot in the STM images of the arrayed device hydrogen lithography patterns (see Supplementary Fig. 2.), we identify single dangling bonds, valid and invalid dimer sites for P incorporation sites using red dots, green ellipses, and red ellipses, respectively. Previous studies[2–4] have shown that the number of incorporated P atoms at STM-patterned quantum dot can be estimated by counting the number of hydrogen-desorbed dimers that are valid for P incorporation. We have carried out previous studies to verify the dopant atom incorporation probability in patterned devices by comparing the STM lithography to few atom quantum dot spectroscopy as described in more detail in Supplementary Note 3. Having at least three adjacent H-desorbed dimers within the same dimer row

is a necessary condition to incorporate one P dopant into the surface silicon lattice. We define the best estimate of the number of incorporated P atoms at each site by requiring three contiguous dimers which are then counted as capable of incorporating a single P dopant. The upper bound of our estimate of the number of incorporated P atoms is set by 25% of the number of dangling bonds sites within allowed dimers, according to the 0.25 monolayer of P incorporation density in saturate-doped Si:P δ-layers.[5] To estimate the lower bound of incorporated P atoms we follow Fuchsle *et al.*,[4] where it is found that, with a saturation dose of phosphine gas at room temperature, the P incorporation density in nm scale desorbed areas decreases to 0.09 monolayer of contiguous desorbed areas within the quantum dot pattern due to competition for dangling bond sites to lose H atoms from absorbed PH_x (x = 1, 2) during incorporation. These estimates agree with our independent evaluation of incorporation probabilities using few dopant atom transistors as described in Supplementary Note 3. We round the estimated lower and upper bound dopant numbers to their nearest integers. Supplementary Table 1 lists the best estimated numbers of dopants at each quantum dot and the lower and upper bounds of the estimates. While it is possible to count the number of incorporated dopants directly by imaging the quantum dots after P incorporation using STM, we avoid this time-consuming step to minimize the exposure of the reactive patterned device to contamination before epitaxial encapsulation as well as the risk of unintentional tip-surface interactions that could introduce atom-scale contaminations and defects.

Site Index	Estimated Number of Dopants								
	First Array			Second Array			Third Array		
	Lower Bound of Estimate	Best Estimate	Upper Bound of Estimate	Lower Bound of Estimate	Best Estimate	Upper Bound of Estimate	Lower Bound of Estimate	Best Estimate	Upper Bound of Estimate
D11	1	2	3	1	3	4	2	3	5
D12	1	2	3	2	3	5	2	3	5
D13	0	2	4	1	2	3	2	3	5
D21	0	1	1	1	2	3	2	3	6
D22	1	2	3	1	3	4	2	3	6
D23	1	2	3	1	2	3	0	1	2
D31	1	2	3	2	3	5	2	3	5
D32	0	1	1	1	2	3	2	3	5
D33	1	2	4	1	2	3	1	2	3

Supplementary Table 2. Estimated number of incorporated P atoms at each quantum dot. The ranges of estimates are based on analysis in Supplementary Fig. 2. See descriptions in Supplementary Note 2 for the criteria of the lower bound, the upper bound, and the optimum of the estimates in this table.

Dopant Number	E_b	U_i
1P	-47	44
2P	-70 ± 10	45 ± 10
3P	-81 ± 6	46 ± 7

Supplementary Table 3. Binding energy E_b and addition energy U_i (for the relevant charge-neutral to negatively charged state transitions near the Fermi level) of few-P cluster quantum dots. The numbers in the table are summarized from Weber, et al.[6] E_b values are with respect to the conduction band edge. The variations for 2P and 3P clusters represent one sigma (70%) in cluster configuration distributions.[6]
We account for the variations in the best-estimate numbers of dopant atoms per site (Supplementary

Table 2) by adopting the corresponding binding energy (average value) as listed in the second column. The charging energy values in the last column are used to choose the dot size in the capacitance model so that the calculated on-site charging energy (See Supplementary Table 1) matches (within a few meV) to the atomistically calculated charging energy values.

Supplementary Note 3. Characterizing an individual few-dopant quantum dot

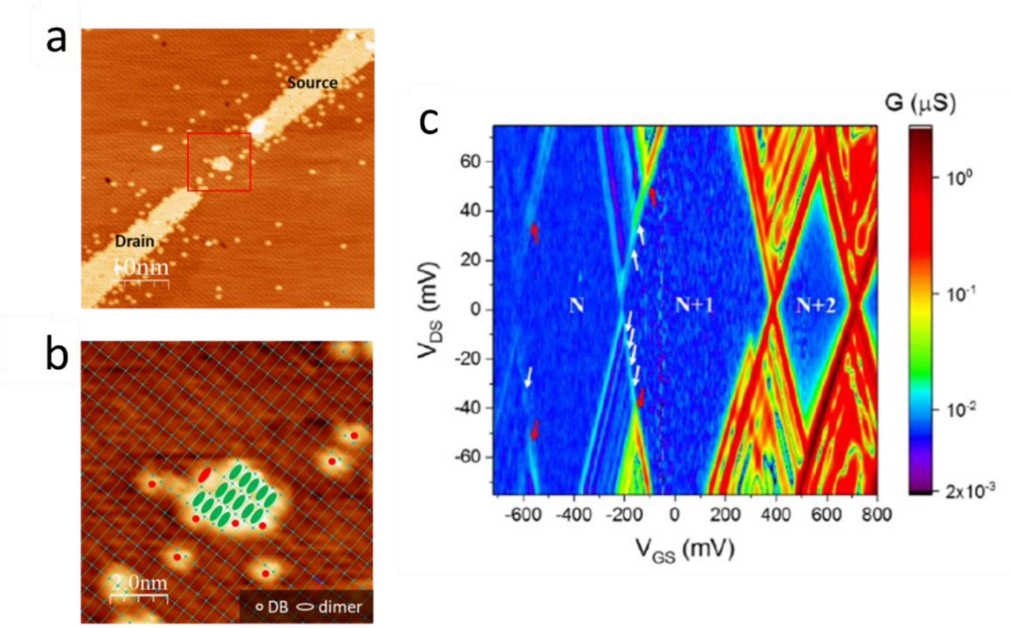

d

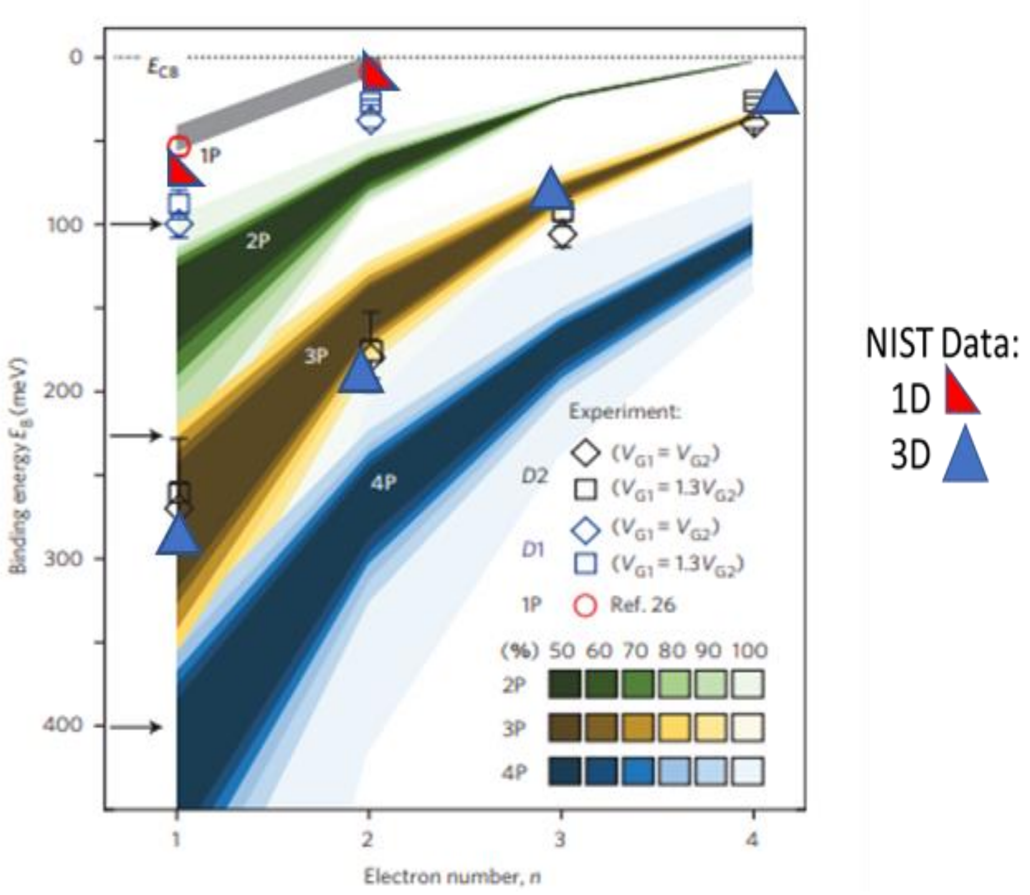

Supplementary Fig. 3. An example of few-dopant quantum dot spectroscopy. (a) STM images of the central region of the few-dopant quantum dot after hydrogen lithography, but before PH_3 dosing. (b) Close-up STM image of the H-desorbed quantum dot region. The number of exposed Si dangling bonds (DB) and dimers can be counted by overlaying the Si (100) 2x1 surface reconstruction lattice grids with the STM images after hydrogen lithography. The allowed and forbidden P incorporation sites are highlighted in green and red respectively. (c) the low-temperature (T=4 K) differential conductance charge stability diagram. As indicated by arrows at the $N \leftrightarrow N$+1 and N-1$\leftrightarrow N$ transitions, there appears symmetric resonant tunneling features at positive and negative biases, indicating approximately equal tunnel coupling between the dot and the drain and source leads. The occupation number of the dot is expressed using an integer N. (d) Following the method as described by Weber and co-workers,[6] we extract the binding energy spectrum of the few-dopant quantum dot from (c) and overlay the extracted binding energy levels (blue and red triangles) on the experimental and theoretical binding energy

spectrum previously published by Weber and co-workers.[6] The overlay indicates that there are 3 dopant atoms incorporated in the dot shown in (b).

Supplementary Note 4. Impact of hopping, interactions, and disorder on simulated addition energy spectrum and charge stability diagrams

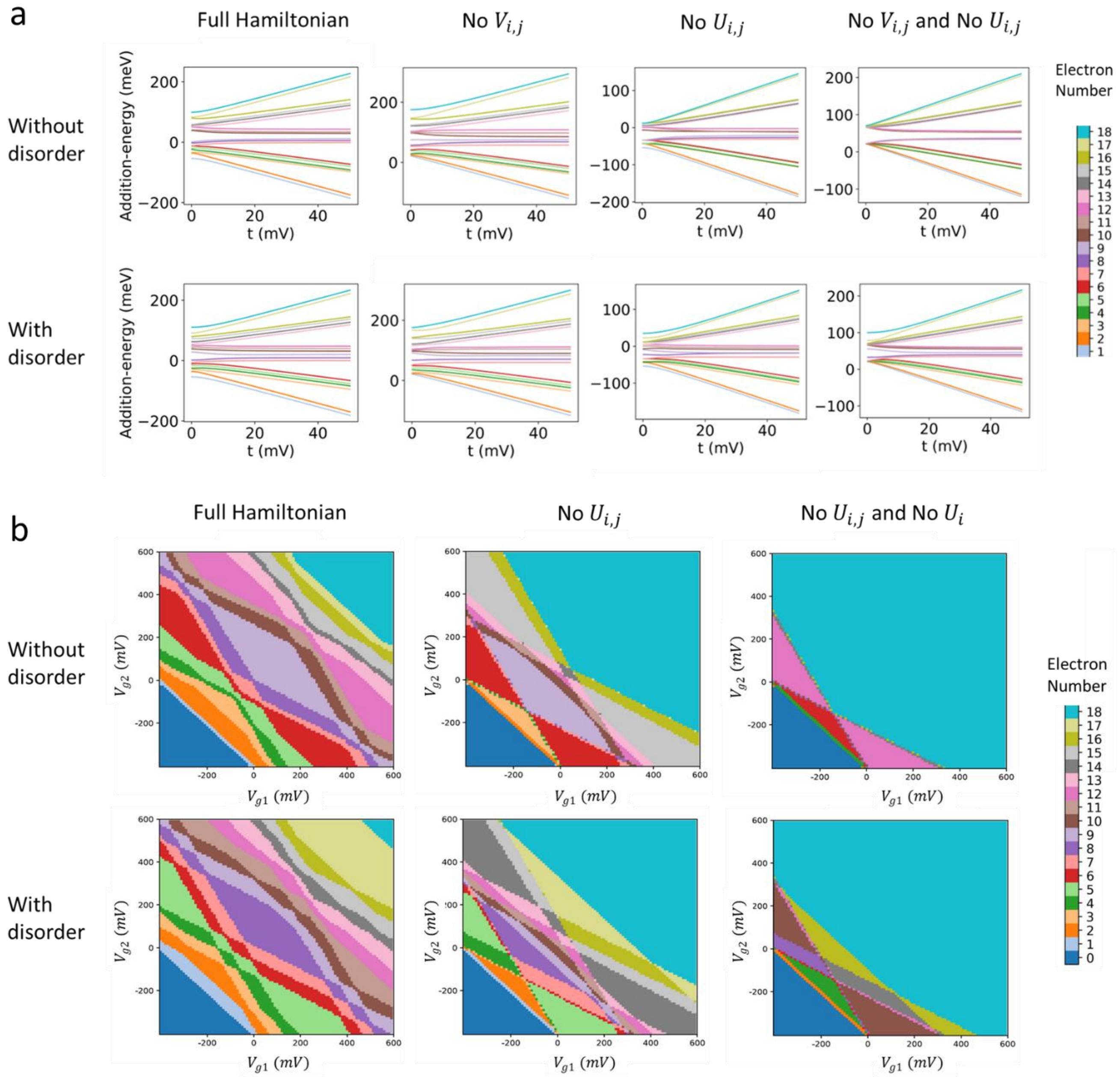

Supplementary Fig. 4. Impact of hopping, interactions, and disorder on simulated addition energy spectrum and charge stability diagrams. (a) Simulated charge addition energy spectrum of the third

array as a function of hopping amplitude (t) with all leads and gate electrodes at zero ground potential. The impact of long-range electron-electron Coulomb repulsion ($U_{i,j}$) and long-range electron-ion core Coulomb attraction ($V_{i,j}$), on the charge addition energy spectrum are illustrated by comparing plots in the first column calculated using the full Hamiltonian (Equation 1 in the main text) and plots in the subsequent columns where $V_{i,j}$ and/or $U_{i,j}$ are turned off. The panels in the upper row assume identical three-dopant quantum dots with no variation in binding energy, while the lower row of panels takes into account the estimated disorder in binding energy (see Supplementary Tables 2 and 3). We have calculated with different sets of disorder configurations and found that they do not alter the qualitative features as shown in the lower panels in (a). The 18 addition energy levels correspond to a total of 18 excess electrons modeled in the 3x3 array system. (b) Simulated charge stability diagrams of the third array illustrating the impact of long-range and on-site electron-electron Coulomb interactions. Diagrams in the panels of the first column are calculated using the full Hamiltonian; diagrams in the second and the third column panels are calculated respectively by turning off the long-range electron-electron interaction ($U_{i,j}$) only and by turning off both the long-range and on-site electron-electron interactions ($U_{i,j}$ and U_i) all together. Following the simulation conditions in (a), diagrams in the upper row of panels and lower row of panels are calculated assuming an ideal and a disordered array configuration, respectively.

In Supplementary Fig. 4(a), we illustrate the impact of hopping amplitude (t), long-range electron-electron repulsion ($U_{i,j}$), electron-ion Coulomb attraction ($V_{i,j}$), and disorder in binding energy on the upper and lower Hubbard minibands in the simulated charge addition spectrum. The ground state addition energy levels for adding the $\mathrm{N^{th}}$ electron onto the array, $\mathrm{E_{add}^0}$ are defined as

$$\mathrm{E_{add}^0(N) = E_N^0 - E_{N-1}^0}$$

Supplementary Eq. (8)

here $\mathrm{E_N^0}$ is the N-electron ground eigen-energy of the array. The upper panels in Supplementary Fig. 4(a) assume no disorder induced variation in the binding energy of lattice sites (identical three-dopant quantum dots) and hopping amplitude. The hopping amplitude is swept from the weak tunnel coupling regime where t<<U to the intermediate/strong tunnel coupling regime where t ~ U. At t=0 and in the absence of inter-site Coulomb interactions, the Mott gap equals the charging energy at each quantum dot. In this case, transport through the array is in the classical Coulomb blockade regime because the charge addition spectrum is almost entirely determined by the on-site charging energy (U_i) of each identical quantum dot. At t>0 and in the presence of inter-site Coulomb interactions, both hopping and inter-site interactions broaden the Hubbard bands and suppress the Mott gap at half-filling. In this case, transport through the array is in the collective Coulomb blockade regime[7,8] because the charge addition spectrum is determined by the combined effects of the on-site and inter-site interactions within the array. Similar computational observations have been previously reported by Le, Fisher, and Ginossar[9] in dopant-based arrays of similar size.[9] In this study, we extend previous efforts in simulating dopant arrays by including two experimentally defined in-plane gates in the extended Hubbard model and explore their impact on the charge distributions and addition energy spectrum in the array.

In Supplementary Fig. 4(b), we illustrate the impact of long-range and on-site electron-electron Coulomb repulsion ($U_{i,j}$ and U_i) on the simulated charge stability diagrams. As expected, the absence of electron-electron interactions reduces the charge stability regions and the overall span of the charge

stability spectrum over the gate-gate space. Eliminating the many-body interaction effects (no $U_{i,j}$ and no U_i) all together results in charge stability diagrams of the single-particle energy spectrum in the noninteracting regime. The dominate charge stability regions in the noninteracting regime are determined by the overall chemical potential landscape within the array, a combined effect of on-site binding energy, long-range electron-ion Coulomb attraction, and electrostatic potential from electrodes.

Supplementary Note 5. Effects from decreasing the lattice constants within the array

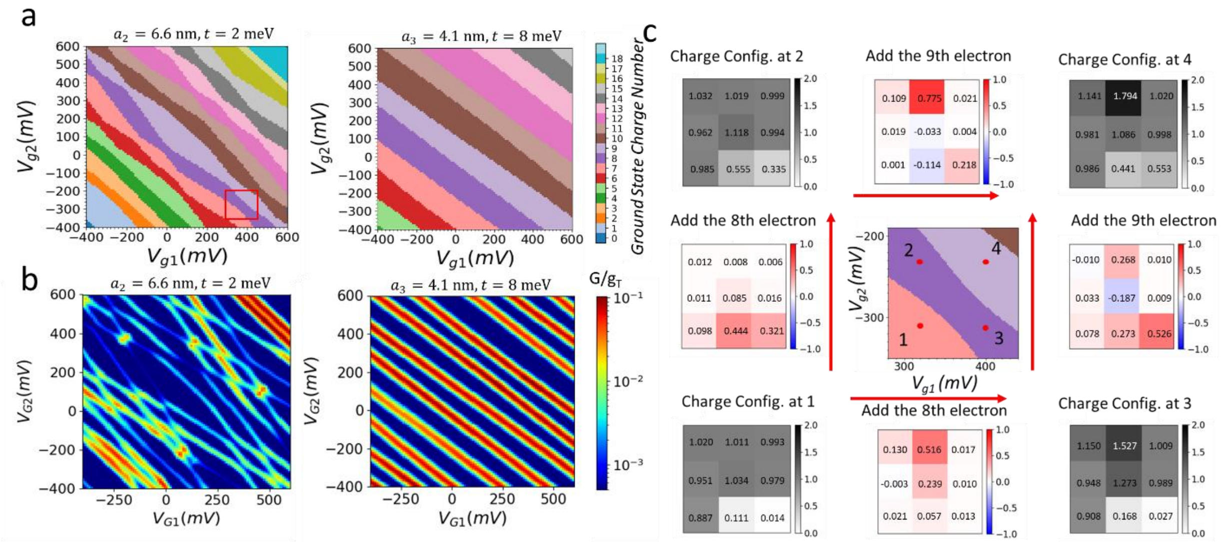

Supplementary Fig. 5. Effects from decreasing the lattice constants within the array. (a) Numerically simulated charge stability diagrams with decreased lattice constants and increased hopping amplitudes. (b) Numerically simulated conductance maps of the corresponding charge stability diagram in (a) at $k_B T = 1meV$. (See Methods) Both sets of diagrams in (a) and (b) share the same color bars on the right. (c) Schematic illustration of simulated eigenstate charge distributions and single charge addition for the second array charge stability diagram region as highlighted by the red box in the left diagram in (a). Note the significant increase in electron delocalization as the lattice constants are reduced. The ground state charge distribution and charge addition plots following the same convention as described in the main text Fig. 3c. For the right and left diagrams in (a) and (b), the input parameters of capacitance matrices and estimates of the numbers of dopants per site are based on the device geometries of the first and second arrays, respectively (see main text Fig. 2a and 2d, and Supplementary Notes 1 and 2).

Supplementary Note 6. Impact of disorder on simulated charge stability diagrams for the third array

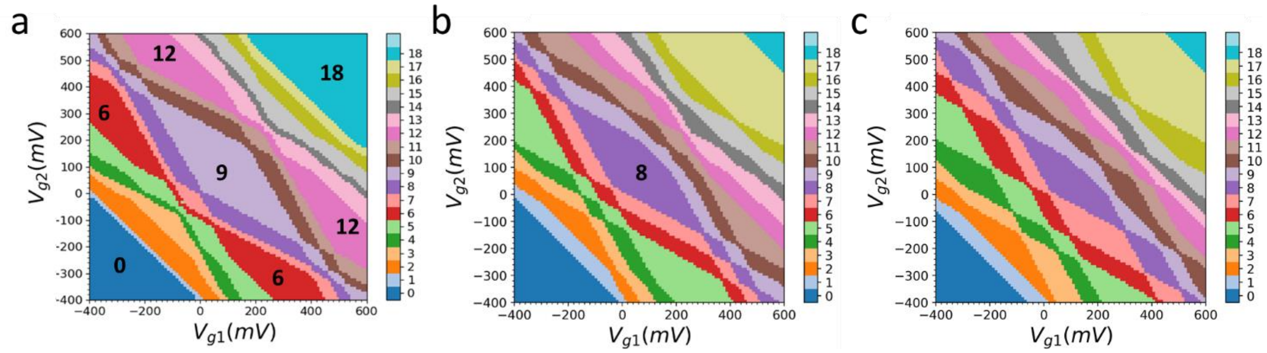

Supplementary Fig. 6. Understanding the impact of disorder and gate potential gradient on the ground eigenstate charge stability diagram. The simulated charge stability diagram in (a) is based on an idealized array with no disorder; all lattice sites are of identical three-dopant cluster quantum dot (binding energy $\mathrm{E_b} = -81\ \mathrm{meV}$) and all nearest-neighbor hopping amplitudes $\mathrm{t} = 0.5\ \mathrm{meV}$. Charge occupation numbers at representative regions are highlighted in the diagram. When the two in-plane gates are tied and sweeping together along the diagonal direction, a finite analog Mott gap is observed at half-filling (occupation = 9), separating the lower Hubbard band from the upper Hubbard band. When a differential voltage is applied across the two in-plane gates and swept from zero to a large differential value, the gap at half-filling closes, and two other prominent addition-energy gaps at one-third (occupation = 6) and two-thirds (occupation = 12) filling opens. This process occurs due to the lowering of the chemical potential of the first or the third rows of lattice sites causing the electrons to energetically favor doubly occupying those rows and overcoming the on-site charging energy (U), as opposed to half-filling of the entire array. In (b), we introduce disorder in the number of dopant atoms per site based on the best estimates from experimental lithographic patterns shown in the main text Fig. 1c and Supplementary Fig. 2: one dopant at D23 ($\mathrm{E_b} = -47\ \mathrm{meV}$), two dopants at D33 ($\mathrm{E_b} = -70\ \mathrm{meV}$), and three dopants for the rest of the dots ($\mathrm{E_b} = -81\ \mathrm{meV}$). Because of the higher energy cost to add the first electron onto D23, the central Mott-like gap along the diagonal direction now appears with charge number 8. In (c), the dopant number at each site is the same as those in (b), however, random noise from a Gaussian distribution with zero mean and standard deviation of 10 meV has been added to $\mathrm{E_b}$to gain a better idea about the effect of variations in the number of dopants at each site. Note that (c) is not averaged over disorder realizations; it represents a typical case in which there is significant variation in $\mathrm{E_b}$ across the array. Along the diagonal direction, the added disorder further broadens the upper and lower Hubbard bands and suppresses the gap.

Supplementary Note 7. Eigenstates, charge addition, and resonance at avoided crossings

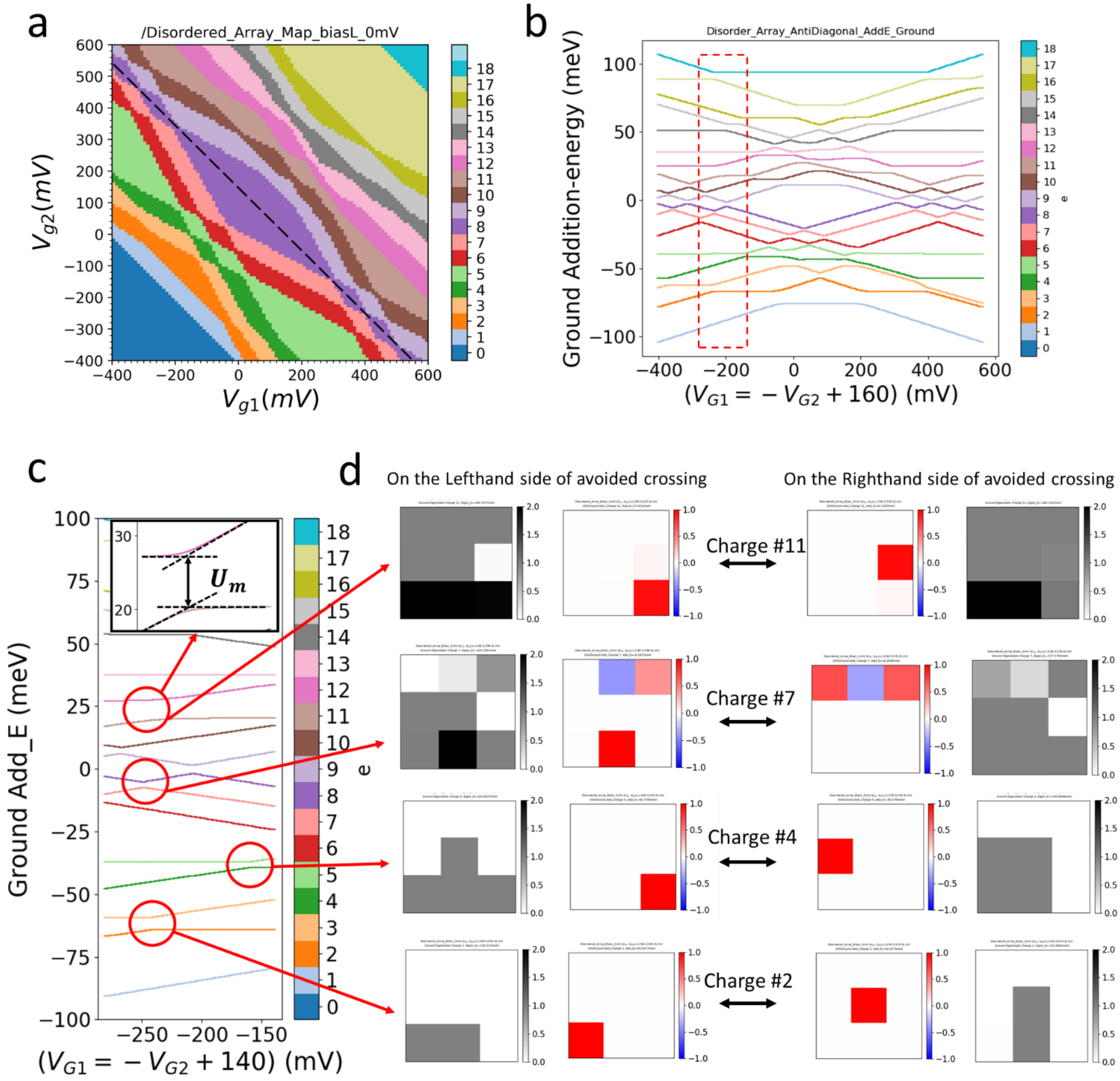

Supplementary Fig. 7. Simulated charge addition energy spectrum and charge distribution at avoided crossings. (a) Simulated ground eigenstate charge stability diagram of the third 3x3 array, reproduced from Fig. 3a in the main text. (b) calculated ground-state charge-addition-energy level spectrum along the dashed line in (a). See Supplementary Note 4 for the definitions of the ground-state charge-addition-energy levels. The addition energy spectrum is plotted with respect to the source/drain leads' Fermi level, which sets the zero-energy level in the addition energy spectra for adding electrons from the source/drain leads. The 18 electron addition energy levels correspond to a total of 18 excess electrons

that can be added to the 3x3 array at gate voltage conditions along the dashed line in (a). We emphasize the apparent similarity between the charge addition boundaries in the gate-gate charge stability map and the charge addition energy levels in the addition energy spectrum, manifesting the same charge addition response to the chemical potential gradient across the array in the gate-gate direction; the charge addition energy is flat versus the potential gradient when an electron is added to the middle row sites; the charge addition energy increases (decreases) with increased negative voltage on the upper gate when an electron is added to the upper (lower) row sites. (c) Charge occupation configurations on the left and right sides of selected avoided crossings in the addition energy spectrum region that are highlighted in (b). The charge configurations become resonant at the avoided crossing. As discussed in (b), it is energetically favorable for an electron to be added to different lattice sites on the left and right sides of an avoided crossing, as indicated by the different addition energy slopes on either side. The energy separation at an avoided crossing in the addition energy spectrum characterizes $(\mathrm{U_m} + 2\mathrm{t}')$, where $\mathrm{U_m}$ is the mutual charging energy between the two involved charge addition distributions and t' is the resonant tunnel coupling between the two charge distribution configurations. The subplot in (c) illustrates that the $\mathrm{U_m}$ contribution at an avoided crossing can be obtained by extrapolating the linear sections on both sides of the avoided crossing, in analogy to standard practices in double-dot systems. Since the system is in the strong interaction regime $(\mathrm{U_i}, \mathrm{U_{i,j}} \gg \mathrm{t})$, $\mathrm{U_m}$ dominates the avoided crossing separation. (d) Schematic illustration of eigenstate charge distributions and single charge addition at the left and right sides of the avoided crossings as highlighted in (c). Note, the zero addition energy level in (c) corresponds to the Fermi level in the source and drain leads. Taking charge addition #7 as an example, the gray scale plots represent the charge distributions of many-body ground states when there are 7 electrons in the array. The red-blue plots represent the change in charge distributions when adding the 7th electron onto the array and the system changes from a 6-electron ground state to a 7-electron ground state. At the avoided crossings when adding charges #2, #4, and #11, the involved charge addition sites are single lattice sites, and the avoided crossing separation is large (small) when the two resonant sites are close to (far apart from) each other. At the avoided crossing for adding charge #7, due to finite hopping amplitude, the addition of a single electron leads to changes in occupation at multiple lattice sites. The resonance between the two coupled many-body charge configurations is a manifestation of the complex many-body interactions, such as the mutual charging energy (long range Coulomb interactions) and tunnel coupling, between many-body states in the array system.

**Representative Avoided-crossings in Supplementary Fig. 7.**	**Addition Energy at the Avoided-crossing (meV)**	**Charge-addition sites**		**Spatial distance between the two charge-addition distributions**	**Mutual charging energy (U_m) between the two charge-addition distributions (meV)**	**2 × Tunnel coupling $(2t')$ between the two charge-addition distributions (meV)**
		On the Lefthand side of avoided crossing	On the righthand side of avoided crossing			
Add Charge #2	4.99(1)	D31	D22	1.4×a	4.82(3)	~0.17
Add Charge #4	2.16(4)	D33	D21	2.2×a	2.14(4)	~0.02
Add Charge #7	2.22(5)	D32	D11, D12, D13	~2×a	2.19(5)	~0.03

Add Charge #11	8.06(1)	D33	D23	a	6.74(3)	~1.30

Supplementary Table 4. Quantitative analysis of the charge addition energies from the avoided crossings in Supplementary Fig. 7 (c). In analogy to single-electron resonant tunneling in a double-dot system, the addition energy separation at an avoided crossing in a 3x3 array with two in-plane gates is characterized by $(U_m + 2t')$, where U_m is the mutual charging energy (long-distance e-e repulsion) between the two charge addition distributions (see the red-blue charge distribution plots in Supplementary Fig. 7(d)) that are on resonance at the avoided crossing, and t' is the resonant tunnel coupling between them. The listed addition energies (2nd column) at the avoided crossings are extracted from the numerically simulated addition energy spectrum with hopping t = 0.5 meV, as shown in Supplementary Fig. 7(c). The U_m at an avoided crossing is illustrated in the subplot in Supplementary Fig. 7(c). The U_m values (6th column) at the avoided crossings are extracted from numerically simulated addition energy spectrum with hopping t = 0 meV. The uncertainty in the least significant digit is limited by the x-axis (gate voltage) resolution in the numerical simulation. The $\mathbf{2t'}$ values in the last column are estimated as the difference between the addition energy and U_m at the avoided crossings. The numbers support the observation that the mutual charging energy is inversely proportional to the spatial distance between the charge occupation configurations, while the resonant tunneling rate is exponentially dependent on this spatial distance.

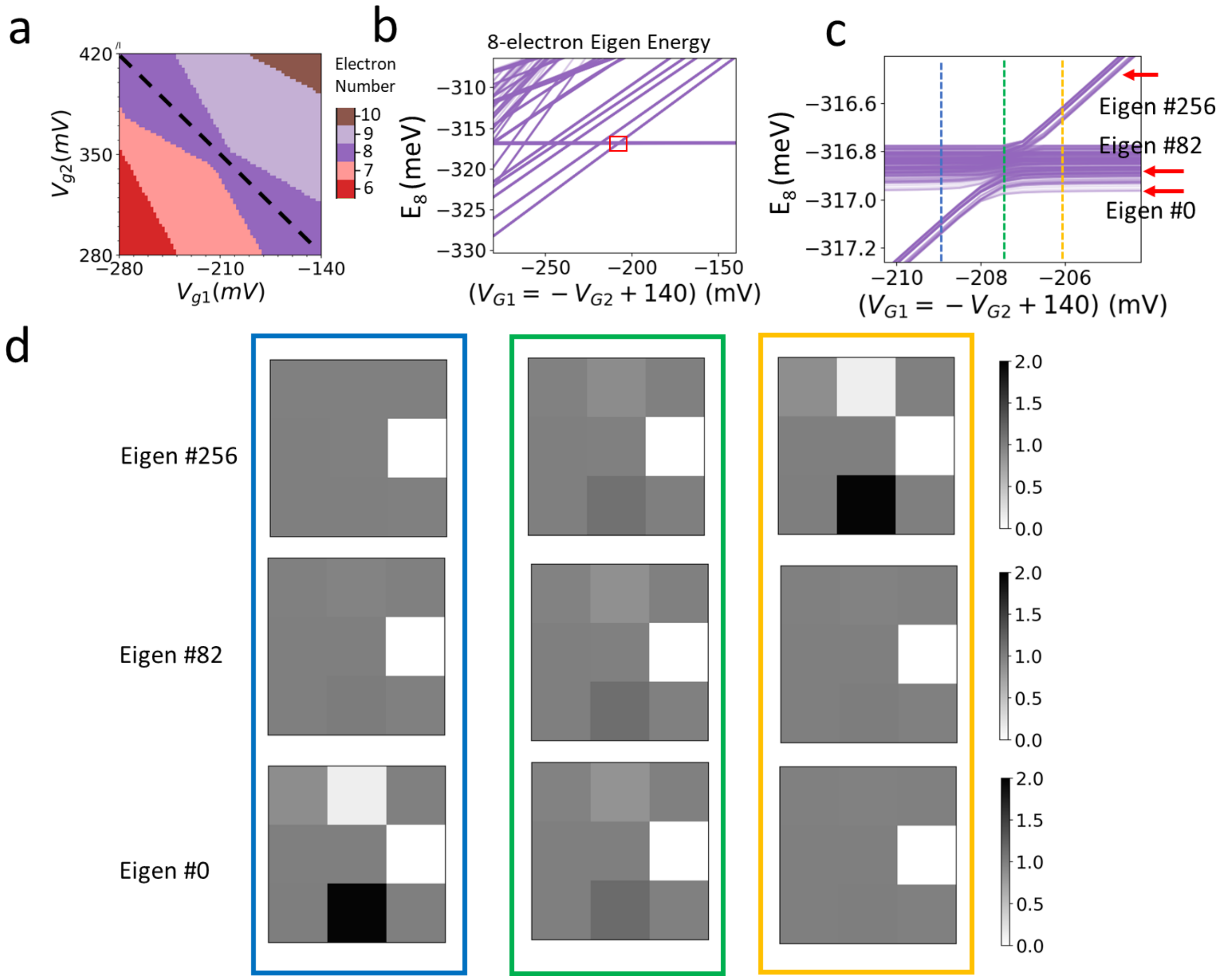

Supplementary Fig. 8. Eigen-energy spectrum and many-body charge distributions. (a) Avoided crossing region reproduced from Fig. 3a in the main text. (b) Simulated eigenenergy spectrum for occupation = 8 eigenstates along the dashed-line detuning axis in (a). (c) Close up eigenenergy spectrum at the red circle in (b). The eigenenergy lines are plotted with 80% transparency, and the color intensity reflects the level of degeneracy. The eigenenergy levels bundle into minibands of highly degenerate or near-degenerate eigenstates. (d) The charge distribution of selected eigenstates at different locations along the detuning axis as highlighted in (c). Charge distribution configurations are nearly identical for eigenstates within the same miniband. Moving along the detuning axis, the ground state alters its charge distribution. The energy separation at the avoided crossing shown in (c) is determined by the tunnel coupling t′ between the two distributions whose eigenstates are hybridizing at the avoided crossing.

Supplementary Note 8. Characterizing addition energy spectrum in the second and third arrays

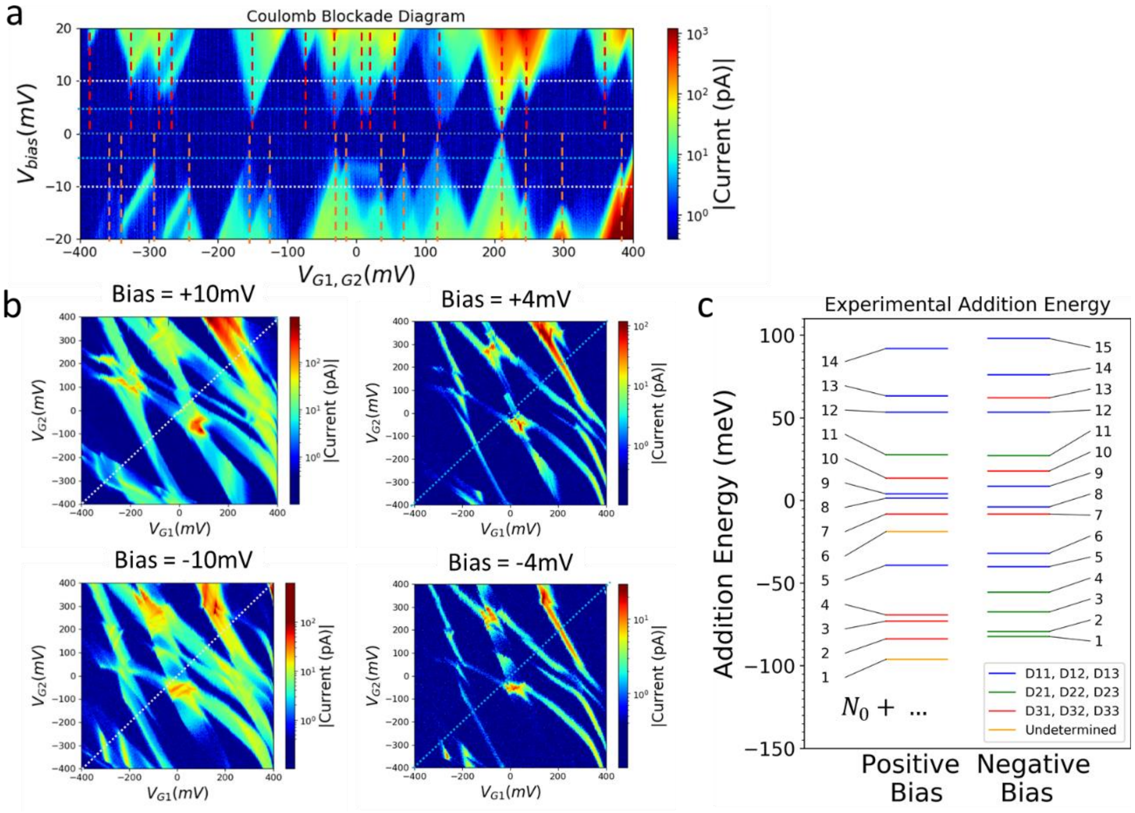

Supplementary Fig. 9. Extracting addition energy levels in the third array using the measured Coulomb blockade diagrams. (a) Coulomb blockade conductance diagram taken along the 45-degree diagonal axis that is marked by dashed lines in (b). Horizontal dashed lines in (a) correspond to the bias voltage level of (b). The vertical dashed lines in (a) mark charge addition positions on the gate-voltage axis where conductance via the addition electrons becomes visible at finite biases. (b) Conductance gate-gate maps that are measured at representative bias voltages, +10 mV, +4 mV, -10 mV, and -4 mV, respectively. Maps are plotted in log-color scale to show conductance features of small amplitudes. (c) Charge addition energy spectra extracted by scaling the measured charge addition levels using properly chosen gate lever arms. The slopes of the charge addition lines in the gate-gate maps allow us to determine whether the conducting electron is added onto sites in the upper, middle, or lower row of the array, and to choose proper gate lever arms for converting charge addition positions on the gate voltage axis to energy levels in the addition energy spectrum. N_0 represents the initial number of excess electrons in the array.

Experimental determination of the charge addition spectrum in a quantum dot array is a prerequisite for more complex measurements and control of many-body states in the array. We use the positions of Coulomb oscillation peaks in the measured Coulomb blockade diagram, as marked by vertical dotted lines in Supplementary Fig. 9 (a), to extract the addition-energy spectrum along the 45-degree diagonal axis in the gate-gate voltage parameter space. To choose the proper gate lever arms to convert measured charge addition levels from the gate voltage axis to energy space, we determine the average

gate lever arm ($\alpha_r = \left(\sum_{i\in r} \alpha_{G1,i} + \alpha_{G2,i}\right)/3$ for adding an electron to sites in row r) according to the gate-gate slope of its charge addition boundary. We plot the charge addition energy spectra in Supplementary Fig. 9 (c), for both positive bias and negative bias conditions. The color of the energy levels corresponds to the gate-gate slope of the conduction lines, which also corresponds to the row of sites where the conducting electron is added. The differences in the extracted addition energy levels between a positive bias condition and a negative bias condition result from the differences in specific atomic configurations along the source/drain direction. We note that the absolute numbers of excess electrons in this device are unknown; however, the assigned charge numbers in Supplementary Fig. 9 (c) do not affect the underlying physical phenomena in this work.

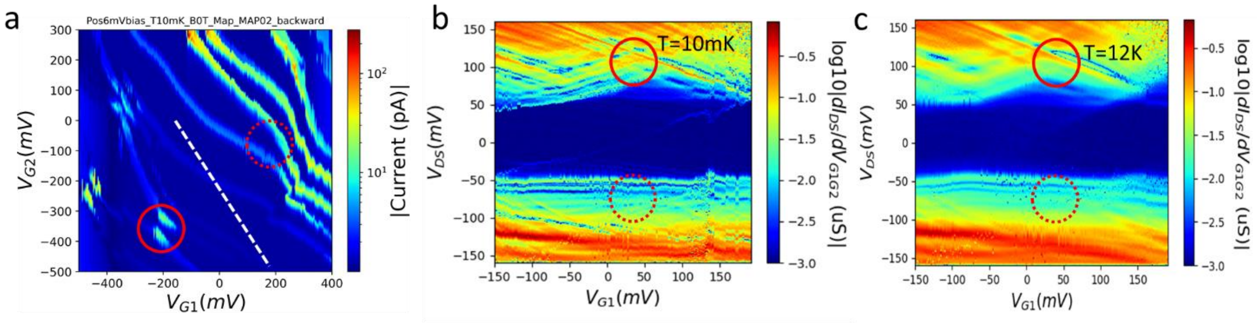

Supplementary Fig. 10. Characterizing the addition energy spectrum in the second array. (a) Gate-gate map of conductance through the array measured at $\mathrm{V_{bias}} = 6\ \mathrm{mV}$ and at the base temperature of the dilution refrigerator ($\mathrm{T} = 10\ \mathrm{mK}$). (b) (c) Finite bias differential conductance spectroscopy taken from the cut along the dashed line in (a), and measured at T = 10 mK (b) and T = 12 K (c), respectively. When doing a transport measurement, a large positive bias at the drain lead (with source lead grounded) pulls down the chemical potential in the drain lead, and individual electrons propagate through the lower Hubbard band within the bias window. Therefore, the differential conductance spectrum at positive (negative) bias in (b) represents the addition energy spectrum of the lower (upper) Hubbard band. The addition-energy levels in the measured lower Hubbard band section are detuned by the differential gate-gate voltages and exhibit crossings of addition energy levels (solid circle) in (b) and (c), which corresponds to the avoided crossing region (solid circle) in (a).

As discussed in the main text, avoided crossing separations in charge stability diagrams are determined by the effective mutual charging energies U_m and tunnel coupling t' between relevant many-body states. Comparing the simulated diagrams for the second array (left panels in Supplementary Figs. 5a and 5b) with those for the third array (main text Figs. 3a and 3b) we observe the expected larger avoided crossing separations.

Supplementary References

1. Grabert, H. & Devoret, M. H. *Single charge tunneling: Coulomb blockade phenomena in nanostructures*. vol. 294 (Springer Science & Business Media, 2013).
2. Schofield, S. R. *et al.* Atomically Precise Placement of Single Dopants in Si. *Phys. Rev. Lett.* **91**, 136104 (2003).
3. Warschkow, O. *et al.* Reaction paths of phosphine dissociation on silicon (001). *J. Chem. Phys.* **144**, 014705 (2016).
4. Füchsle, M. Precision few-electron silicon quantum dots. (2011).
5. Goh, K. E. J., Simmons, M. Y. & Hamilton, A. R. Electron-electron interactions in highly disordered two-dimensional systems. *Phys. Rev. B* **77**, 235410 (2008).
6. Weber, B. *et al.* Spin blockade and exchange in Coulomb-confined silicon double quantum dots. *Nature Nanotechnology* **9**, 430–435 (2014).
7. Hensgens, T. *et al.* Quantum simulation of a Fermi–Hubbard model using a semiconductor quantum dot array. *Nature* **548**, 70–73 (2017).
8. Stafford, C. A. & Das Sarma, S. Collective Coulomb blockade in an array of quantum dots: A Mott-Hubbard approach. *Phys. Rev. Lett.* **72**, 3590–3593 (1994).
9. Le, N. H., Fisher, A. J. & Ginossar, E. Extended Hubbard model for mesoscopic transport in donor arrays in silicon. *Phys. Rev. B* **96**, 245406 (2017).
